# Andrological prevention in paediatric age: proposal of a new model

**DOI:** 10.1186/1824-7288-41-S2-A70

**Published:** 2015-09-30

**Authors:** Matteo Sulpasso, Christian Chironi, Luigi Greco

**Affiliations:** 1 Paediatric Surgery Unit, Clinica Pederzoli, Peschiera del Garda, 37019, Italy; 2 Family Paediatrician, Italian Paediatric Association (S.I.P.), Italy

## 

Andrological disorders in paediatric age are important: they may cause serious repercussions on normal fecundity [1] and sometimes ease cancer outbreak if not properly and adequately treated. Andrological disorders in paediatric age are: 1) frequently observed disorders, 2) less frequently observed disorders, 3) rare observed disorders. Frequently observed disorders are cryptorchidism, left-sided varicocele, communicating hydrocele and funicular cyst, inguinal hernia, phimosis and hypospadias. Less frequently observed disorders are epididymitis, testicular torsion, microlithiasis, Leydig cells hyperplasia [2] and gynecomastia. Rare observed disorders are gonadal cancer, hypogonadism and ambiguous genitalia. The first *cause* of *male infertility is *left-sided varicocele*; the second is cryptorchidism. Prevention plays a fundamental role: if these disorders are diagnosed and treated precociously, the risk of infertility is considerably reduced*.

The authors suggest a new prevention model based on an Andrological Form to be included in the periodical medical examination by the family paediatrician. If the paediatrician detects an andrological disorder during periodical checks, he/she should refer the patient to the paediatric andrologist or paediatric surgeon. The Andrological Form consists of 11 sections to be filled by the family paediatrician (postnatal, at 6 months, at 1, 5, 10, 11, 12, 13 years and at the age of 14 for the last andrological checks before addressing the patient to the general doctor). The first section consists of the patient's records, weight and height included. Second: patient's pubertal development. Third: testicular volume according to Prader. Forth: testicular position. Fifth section: the presence of varicocele. Sixth: penis disorders (phimosis, hypospadias, balano-preputial adhesions). Seventh: gynecomastia (mono or bilateral and level). Eight: occurrence of other associated genito-urinary disorders. Ninth: previous andrological surgery interventions. Tenth: previous medical therapies. Eleventh: sport practicing. The Andrological form should be filled at the following ages: 1) 6 months (testicular position, hypospadias, peritoneo-vaginal duct disorders); 2) 1 year old (testicular position, hypospadias, peritoneo-vaginal duct disorders); 3) 5 years old (phimosis, balano-preputial adhesions, peritoneo-vaginal duct disorders); 4) 10 years old (pubertal development, left-sided varicocele, gynecomastia); 5) at 11, 12, 13 and 14 years old (left-sided varicocele, pubertal development, gynecomastia).

The periodic compilation of the Andrological Form [3], therefore, allows a precocious identification and treatment of these disorders and male infertility prevention.
